# Hypertrophic Osteopathy Associated with a Prostatic Adenocarcinoma in a Castrated Dog

**DOI:** 10.3390/vetsci9090466

**Published:** 2022-08-30

**Authors:** Françoise A. Roux, Emilie Deseille, Marion Fusellier, Marine Rohel, Julien Branchereau, Jack-Yves Deschamps

**Affiliations:** 1 Emergency and Critical Care Unit, Oniris, Nantes-Atlantic College of Veterinary Medicine, Food Science and Engineering, La Chantrerie, CS 40706, CEDEX 03, 44307 Nantes, France; 2 Nutrition, PathoPhysiology and Pharmacology (NP3) Unit, Oniris, Nantes-Atlantic College of Veterinary Medicine, Food Science and Engineering, La Chantrerie, CS 40706, CEDEX 03, 44307 Nantes, France; 3 Department of Diagnostic Imaging, CRIP, Oniris, Nantes-Atlantic College of Veterinary Medicine, Food Science and Engineering, La Chantrerie, CS 40706, CEDEX 03, 44307 Nantes, France; 4 Department of Biology, Pathology and Food Sciences, Laboniris, Oniris, Nantes-Atlantic College of Veterinary Medicine, Food Science and Engineering, La Chantrerie, CS 40706, CEDEX 03, 44307 Nantes, France; 5 Institut de Transplantation-Urologie-Néphrologie, CHU de Nantes, 30 Boulebard Jean Monnet, CEDEX 1, 44093 Nantes, France

**Keywords:** hypertrophic osteopathy, prostatic adenocarcinoma, paraneoplastic syndrome, prostate, cancer, dog

## Abstract

**Simple Summary:**

The present paper describes a rare case of hypertrophic osteopathy in a castrated dog with prostatic cancer and multiple metastases (lymph nodes, liver, spleen, bones and lungs). Hypertrophic osteopathy is a syndrome characterized by diffuse bone formation along several bones, most often those of the limbs, usually in association with a tumor located in the thoracic cavity (here, pulmonary metastases).

**Abstract:**

A 6-year-old mixed-breed male Papillon dog, castrated at the age of 7 months, presented for work-up of a difficulty walking associated with constipation and urinary incontinence. Ultrasonography and radiography were consistent with a tumor of the prostate and lymph node metastases. An irregular osteoproliferation of the ventral edges of L5–L6–L7 suggested tumor invasion. Periosteal proliferative lesions of the pelvis, the femur, the humerus, the tibia and the calcaneus were consistent with hypertrophic osteopathy. Necropsy and histological examination confirmed the diagnosis of prostatic adenocarcinoma with lymph node, pulmonary, liver and bone metastases, associated with hypertrophic osteopathy.

## 1. Introduction

A prostatic carcinoma is a neoplasm found in older dogs and tends to be more frequent in castrated dogs [1]. They are known to be aggressive and metastasize in 70 to 80% of cases [2]. Hypertrophic osteopathy is a syndrome characterized by diffuse periosteal bone formation along the diaphysis and metaphysis of bones, most often those of the limbs, in association with a neoplastic or, more rarely, an inflammatory lesion, usually located in the thoracic cavity, more rarely in other locations [3]. This paper reports a rare case of hypertrophic osteopathy associated with a prostatic adenocarcinoma and pulmonary metastases in a neutered dog.

## 2. Case Report

A 6-year-old mixed-breed male Papillon dog, castrated at the age of 7 months, was referred for work-up of a difficulty walking associated with a pelvic limb gait abnormality, which had developed gradually over the previous three weeks, associated with constipation and drop-by-drop urinary incontinence for five days. Unilateral epistaxis had been observed by the owners on the day of the consultation.

Physical examination revealed a marked depression and paraparesis. Mucous membranes were pale. The popliteal lymph nodes were enlarged, and there was severe swelling of the distal pelvic limbs, with pitting edema. The ventral abdominal surface was covered with an extensive hematoma. A distended bladder was noted on abdominal palpation. The dog was tachypneic; auscultation revealed increased expiratory sounds. Pressure on the lumbosacral spine was painful, the limbs were flaccid, proprioception absent, the withdrawal reflex was present but decreased, and sensation was absent in the caudal region (pelvic limbs, perineum, tail), suggesting a spinal cord injury posterior to the fourth lumbar vertebra. The femoral pulse was palpable. A complete blood count (CBC) revealed a marked normochromic normocytic hyporegenerative anemia (hemoglobinemia (Hb) = 68 g/L; range 120–180) associated with neutrophilic leukocytosis and a marked thrombocytopenia (platelets = 35 × 109/L; range 200–500). The prothrombin time (PT) and partial thromboplastin time (PTT) were normal.

**Figure 1:**
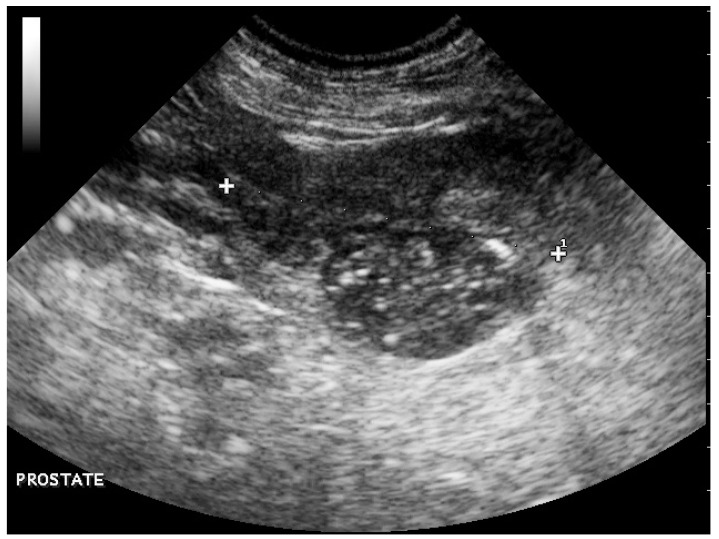
Abdominal ultrasonography showing a small prostate of heterogeneous echogenicity associating hypoechoic foci and hyperechoic calcifications with acoustic shadowing consistent with a tumor of the prostate.

Abdominal ultrasonography revealed a small prostate of heterogeneous echogenicity associating hypoechoic foci and hyperechoic calcifications with acoustic shadowing (Figure 1), which was consistent with a tumor of the prostate. A 2.3-cm-thick mass had effaced the right iliac lymph node, causing compression of the caudal vena cava and aorta; the left iliac and hypogastric lymph nodes were enlarged. These images were compatible with lymph node metastases of prostatic cancer. The ventral edges of the vertebral bodies of L7 and L6 had marked irregularities compatible with bone metastases.

**Figure 2:**
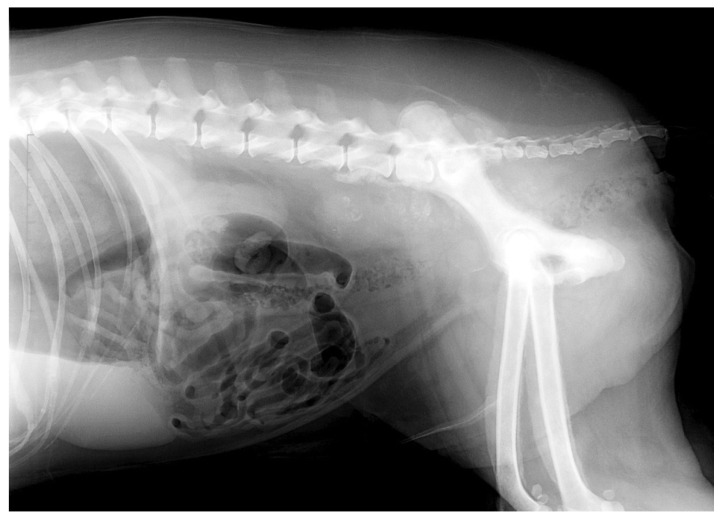
Lateral abdominal radiograph showing a small calcified prostate, a sublumbar mass (probably adenomegaly) that reinforced the diagnosis of prostate cancer with lymph node metastases in a neutered dog. An irregular osteoproliferation of the ventral edges of L5–L6–L7 also suggested tumor invasion. Periosteal proliferative lesions of the pelvis and femurs were consistent with hypertrophic osteopathy.

**Figure 3:**
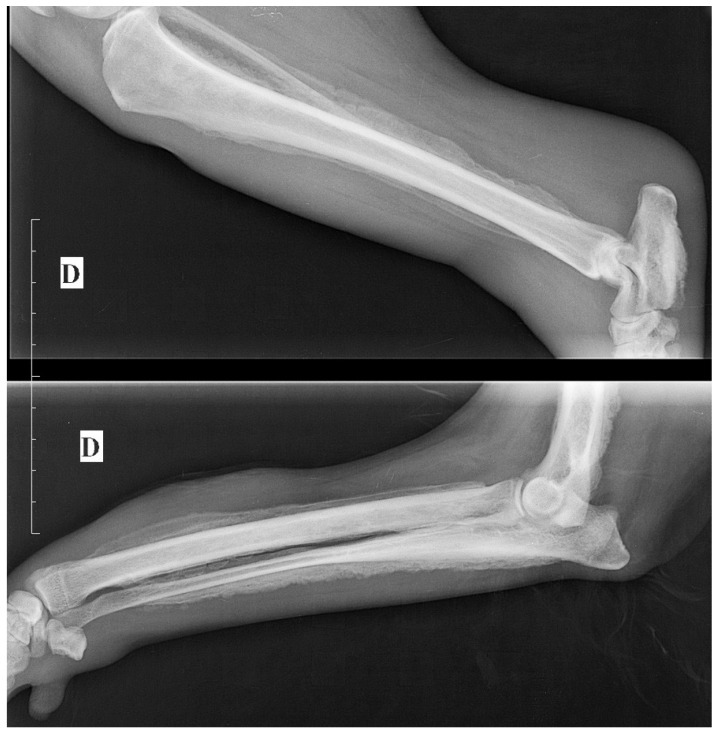
Other lesions consistent with hypertrophic osteopathy observed on the radius, ulna, humerus, tibia and calcaneus.

The lateral abdominal radiograph revealed a small calcified prostate, a sublumbar mass (probably lymphadenomegaly) that reinforced the diagnosis of prostate cancer with lymph node metastases. An irregular osteoproliferation of the ventral edges of L5–L6–L7 also suggested tumor invasion. Periosteal proliferative lesions of the pelvis and femurs were consistent with hypertrophic osteopathy (Figure 2). These characteristic lesions were observed on the radius, the ulna, the humerus, the tibia and the calcaneus (Figure 3). Thoracic radiographs revealed lung metastases and pleural effusion.

A rapid deterioration in the animal’s clinical condition led to a decision to euthanize. On necropsy, the prostate was small and had a 7 mm diameter focus of necrosis in the right lobe. A large paravertebral mass, ventral to L6–L7, 4 cm thick and 8 cm long, with a necrotic center, corresponded to the right medial iliac and lumbar aortic lymph nodes. Multiple metastases from the inguinal and mediastinal lymph nodes were seen, as well as multiple small nodular metastases on the liver, spleen and lungs. The metastatic lung infiltration was associated with a massive pleural effusion in the form of a modified transudate. Recent and extensive bleeding in the abdomen and the jugular region, renal subcapsular petechiae and bilateral epistaxis were explained by thrombocytopenia, probably secondary to disseminated intravascular coagulation (DIC). Periosteal bone production was clearly visible on a longitudinal section of the femur.

**Figure 4:**
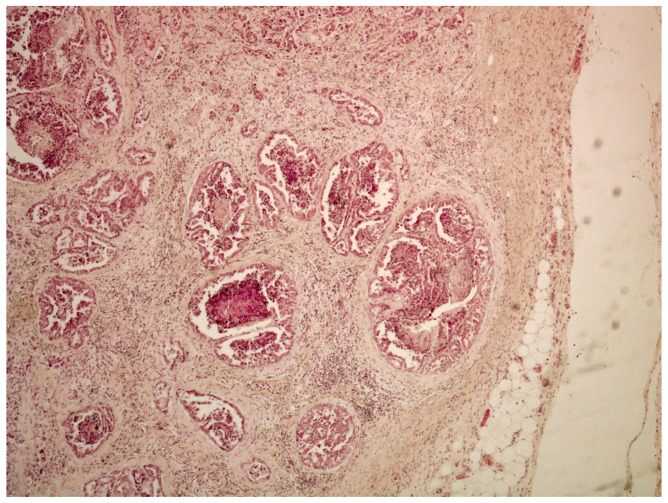
Prostate invaded by a poorly defined unencapsulated tumor, which is densely cellular and infiltrative (HES stain ×40).

**Figure 5:**
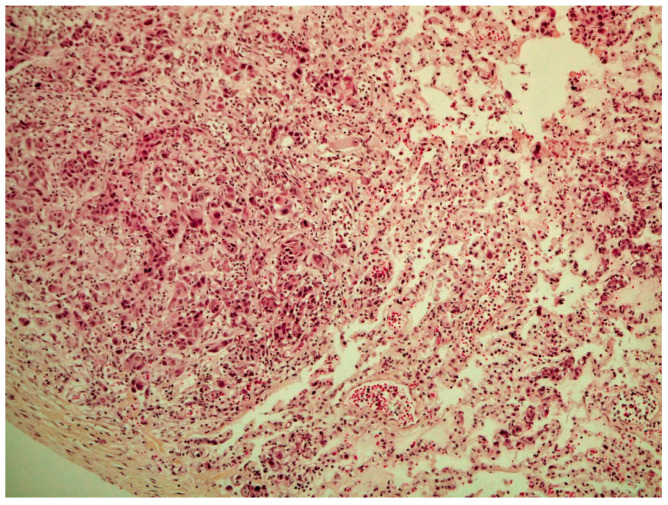
Multiple pulmonary emboli and focal metastatic infiltration of carcinomatous cells (HES stain ×100).

**Figure 6:**
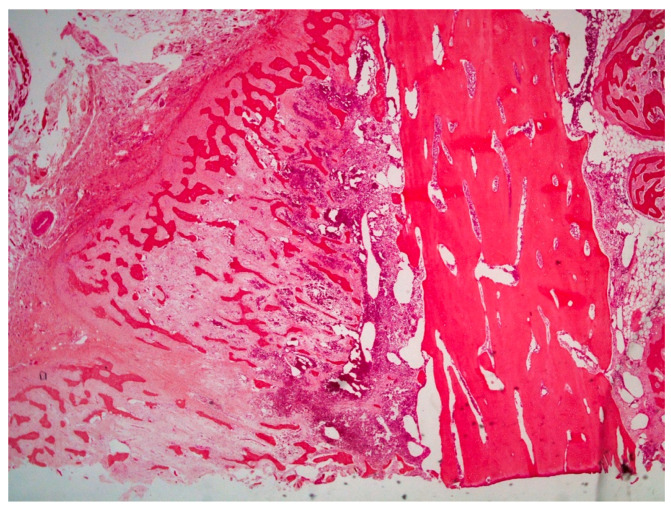
Decalcified longitudinal femur section showing, from right to left, cortex, bony periosteal trabecular proliferation and periosteal reaction characteristic of hypertrophic osteopathy lesions (HES stain ×40).

**Figure 7:**
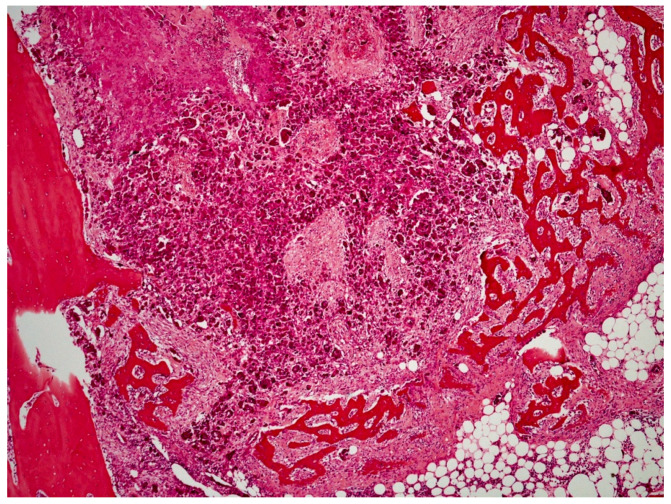
Several foci of carcinomatous cells observed in the medullary cavities of bone and in the periosteal proliferation (HES stain ×40).

On histological examination (HES stain ×40) (Figure 4), the prostate was invaded and replaced by a poorly defined infiltrative unencapsulated and densely cellular tumor. Tumoral population was arranged in multiple lobules of duct-like structures, lined by unique or multiple layers of pleomorphic neoplastic cells, or occasionally in small clusters of neoplastic cells separated by a plentiful stroma response. Metastatic emboli were numerous. Multiple pulmonary emboli and focal metastatic infiltration with carcinomatous cells (HES stain ×100) (Figure 5) confirmed the severe lymphatic and parenchymatous pulmonary tumor extension, which probably caused the hypertrophic osteopathy. A decalcified longitudinal femur section (HES stain ×40) (Figure 6), showed, from right to left, the cortex, perpendicular bony periosteal trabecular proliferation and an exuberant periosteal reaction characteristic of hypertrophic osteopathy lesions. In addition, several concomitant foci of carcinomatous cells were observed in the medullary cavities of the preexisting bone and in the periosteal proliferation (HES stain ×40) (Figure 7). These observations confirmed the diagnosis of prostatic adenocarcinoma with lymph node, pulmonary, liver and bone metastases, associated with hypertrophic osteopathy.

## 3. Discussion

In the dog, the vast majority of prostate tumors are carcinomas [2]. Prostatic adenocarcinomas are highly aggressive malignancies [2]. In a case study, metastases occurred in 89% of dogs; metastases were identified in 100% of castrated dogs [4]. Metastatic sites were, in descending order, the lungs (100% of neutered dogs and 40% of intact dogs), regional lymph nodes (33%), liver (33%), spleen (26%), colon and rectum (22%), bladder (18%), urethra (18%), bones (15%), heart, kidneys, distant lymph nodes and the adrenal glands [4]. The lumbar vertebrae and pelvis are the most common sites for bone metastasis [5]. This dog was euthanized soon after admission; in a retrospective study, 76% of dogs with prostatic adenocarcinoma died or were euthanized within 10 days of diagnosis [4].

Castrated male dogs are at an increased risk of developing prostatic carcinoma [1]. A prostatic lesion with mineralization of the prostate in a dog neutered at a young age is highly suggestive of a tumor; in one study, 100% of neutered dogs with a lesion of the prostate with mineralization had neoplasia [6]. Ultrasound images of the prostate observed in the present case (irregular contours, parenchymal calcifications, presence of hypoechoic areas) are also characteristic of a neoplastic process. The lymph node and bone metastases strengthen this hypothesis.

Hypertrophic osteopathy may be associated with any type of tumor. Periosteal new bone formation is usually confined to the limbs; the distal ends (metacarpus, radius, ulna, metatarsus, tibia) are generally affected first; then the lesions extend proximally. In the present report, lesions of the lumbar vertebrae are more suggestive of a local extension of prostate cancer than hypertrophic osteopathy because of the irregular bone proliferation, in contrast to the lesions observed on the pelvis and the limbs; carcinomatous metastases observed around the bone (preexisting and newly formed) are probably concomitant findings and do not participate in the formation of the hypertrophic bone lesion.

To the author’s knowledge, seven cases of hypertrophic osteopathy associated with prostatic tumors have been reported in dogs. In a case series of 30 dogs with hypertrophic osteopathy, one dog had a prostatic carcinoma [7]. In a case series of 31 dogs with prostatic adenocarcinoma [4], two dogs presented periosteal proliferation suggestive of hypertrophic osteopathy, one on the ventral aspect of the sixth lumbar vertebra, and the other on the femur, tibia and radius. In another report, a periosteal new bone was identified arising from the ventral surface of the fifth, sixth and seventh lumbar vertebrae and on the forelimbs [6]. In the first report of hypertrophic osteopathy associated with prostatic tumors in 1982 [8], and in a more recent report in 2018 [9], no thoracic metastases were observed on postmortem examination. In the most recent case report, published in 2020 [10], in a ten-year-old, neutered male, mixed-breed dog, with a history of lameness, a prostatic tumor with metastasis on the right kidney, the lungs and the mediastinal lymph nodes was diagnosed. On radiographic examination, a lytic bone mass was observed in the left metatarsus, as well as a diffuse proliferative periosteal reaction in several bones of the appendicular skeleton. In the present case, it is very difficult to assess which tumor (primary prostatic carcinoma or lung metastasis) is the primary cause of hypertrophic osteopathy. As seen previously, some cases of prostatic carcinoma with hypertrophic osteopathy were described without any associated thoracic metastasis. There is a possible combined participation of both the primary tumor and lung metastasis in the process.

## 4. Conclusions

In dogs, prostatic carcinomas are aggressive tumors, with high metastasis potential. Hypertrophic osteopathy is mainly associated with lung disease, and usually a primary or secondary tumor. This report describes a rare case of hypertrophic osteopathy in a castrated dog with prostatic adenocarcinoma and multiple metastases (lymph nodes, liver, spleen, bones and lungs). Both the primary prostatic carcinoma and lung metastasis can be responsible for the associated hypertrophic osteopathy in this case.

## Data Availability

All data available on request.
